# Novel Direct Targets of miR-19a Identified in Breast Cancer Cells by a Quantitative Proteomic Approach

**DOI:** 10.1371/journal.pone.0044095

**Published:** 2012-08-30

**Authors:** Mamoru Ouchida, Hirotaka Kanzaki, Sachio Ito, Hiroko Hanafusa, Yoshimi Jitsumori, Seiji Tamaru, Kenji Shimizu

**Affiliations:** 1 Department of Molecular Genetics, Graduate School of Medicine, Dentistry and Pharmaceutical Sciences, Okayama University, Okayama, Japan; 2 Central Research Laboratory, Graduate School of Medicine, Dentistry and Pharmaceutical Sciences, Okayama University, Okayama, Japan; 3 Department of Biomedical Sciences, Cedars-Sinai Medical Center, Los Angeles, California, United States of America; St. Georges University of London, United Kingdom

## Abstract

The miR-17–92 cluster encodes 7 miRNAs inside a single polycistronic transcript, and is known as a group of oncogenic miRNAs that contribute to tumorigenesis in several cancers. However, their direct targets remain unclear, and it has been suggested that a single miRNA is capable of reducing the production of hundreds of proteins. The majority of reports on the identification of miRNA targets are based on computational approaches or the detection of altered mRNA levels, despite the fact that most miRNAs are thought to regulate their targets primarily by translational inhibition in higher organisms. In this study, we examined the target profiles of miR-19a, miR-20a and miR-92-1 in MCF-7 breast cancer cells by a quantitative proteomic strategy to identify their direct targets. A total of 123 proteins were significantly increased after the endogenous miR-19a, miR-20a and miR-92-1 were knocked down, and were identified as potential targets by two-dimensional electrophoresis and a mass spectrometric analysis. Among the upregulated proteins, four (PPP2R2A, ARHGAP1, IMPDH1 and NPEPL1) were shown to have miR-19a or miR-20a binding sites on their mRNAs. The luciferase activity of the plasmids with each binding site was observed to decrease, and an increased luciferase activity was observed in the presence of the specific anti-miRNA-LNA. A Western blot analysis showed the expression levels of IMPDH1 and NPEPL1 to increase after treatment with anti-miR-19a, while the expression levels of PPP2R2A and ARHGAP1 did not change. The expression levels of *IMPDH1* and *NPEPL1* did not significantly change by anti-miR-19a-LNA at the mRNA level. These results suggest that the *IMPDH1* and *NPEPL1* genes are direct targets of miR-19a in breast cancer, while the exogenous expression of these genes is not associated with the growth suppression of MCF-7 cells. Furthermore, our proteomic approaches were shown to be valuable for identifying direct miRNA targets.

## Introduction

MicroRNAs (miRNAs) are endogenous small non-coding single-stranded RNAs, 19 to 23 in length [1], [2]. MiRNAs have been suggested to have oncogenic or tumor suppressive functions through their negative post-transcriptional regulation of protein-coding genes [3], [4]. Many miRNAs exhibit binding activity to the 3′ untranslated region (3′UTR) of target mRNAs as a result of sequence complementarity. It has been estimated that the miRNAs in a whole cell regulate approximately 30% of all protein-coding genes. A single miRNA is also capable of reducing the production of hundreds of proteins [5]. Therefore, by targeting multiple transcripts and affecting the expression of numerous proteins, miRNAs play key roles in cellular development, differentiation, proliferation and apoptosis [6]–[9]. Several studies have also demonstrated that more than 50% of miRNAs are located in cancer-associated genomic regions [10], thus suggesting that miRNAs may also play an important role in cancer.

There are a large number of miRNA targets which have been identified by bioinformatics studies [11]–[13], and many other miRNA targets have been experimentally identified [14]. The target prediction is primarily based on the sequence complementarity between the 5′ end of the mature miRNA and the 3′UTR of the target gene(s). Since there are many cases of both false-positive and false-negative miRNA targets predicted by the current software programs, it is critically important to confirm the miRNA targets by experimental assays [15]. The most extensively used approaches to the target identification of miRNAs include cDNA microarray and real-time PCR-based methods. Considering that the miRNAs are thought to regulate gene expression by translational inhibition, rather than mRNA degradation [1], these methods might thus be problematic when trying to identify direct miRNA targets [16]–[18]. Consequently, a proteomic approach would provide major advantages for identifying direct targets of miRNAs.

The miR-17–92 cluster is one of the best known oncogenic miRNAs, called oncomir-1 [19], which is a polycistronic miRNA encoding miR-17-5p, -17-3p, -18a, -19a, -20a, -19b and -92-1 [20]. These miRNAs are categorized into four separate families according to their characteristic seed sequence: the miR-17 family (miR-17-5p, miR-17-3p, miR-20a), the miR-18 family (miR-18a), the miR-19 family (miR-19a and miR-19b) and the miR-92 family (miR-92-1) [21]. The overexpression of the miR-17-92 cluster has been observed in multiple tumor types [22], [23]. MiR-17-92 is thought to have an oncogenic function in lung cancer and lymphomas [19], [24], whereas the correlation between the expression of miR-17-92 and breast cancer remains unexplored.

In this study, we examined the overexpression of miR-17-92 in MCF-7 breast cancer cells. To identify the direct targets of miR-17-92, we performed profiling of the changes in protein expression that occurred after knocking down miR-17-92 in these breast cancer cells using two-dimensional electrophoresis (2-DE). By global proteomic profiling, we identified 123 putative targets of miR-17-92. In subsequent validation studies, we demonstrated that a subset of these targets were direct targets of miR-19a.

## Results

### The Expression of the miR-17-92 Cluster in MCF-7 Cells, and its Inhibition by an Anti-miRNA-locked Nucleic Acid (LNA)

**Figure 1:**
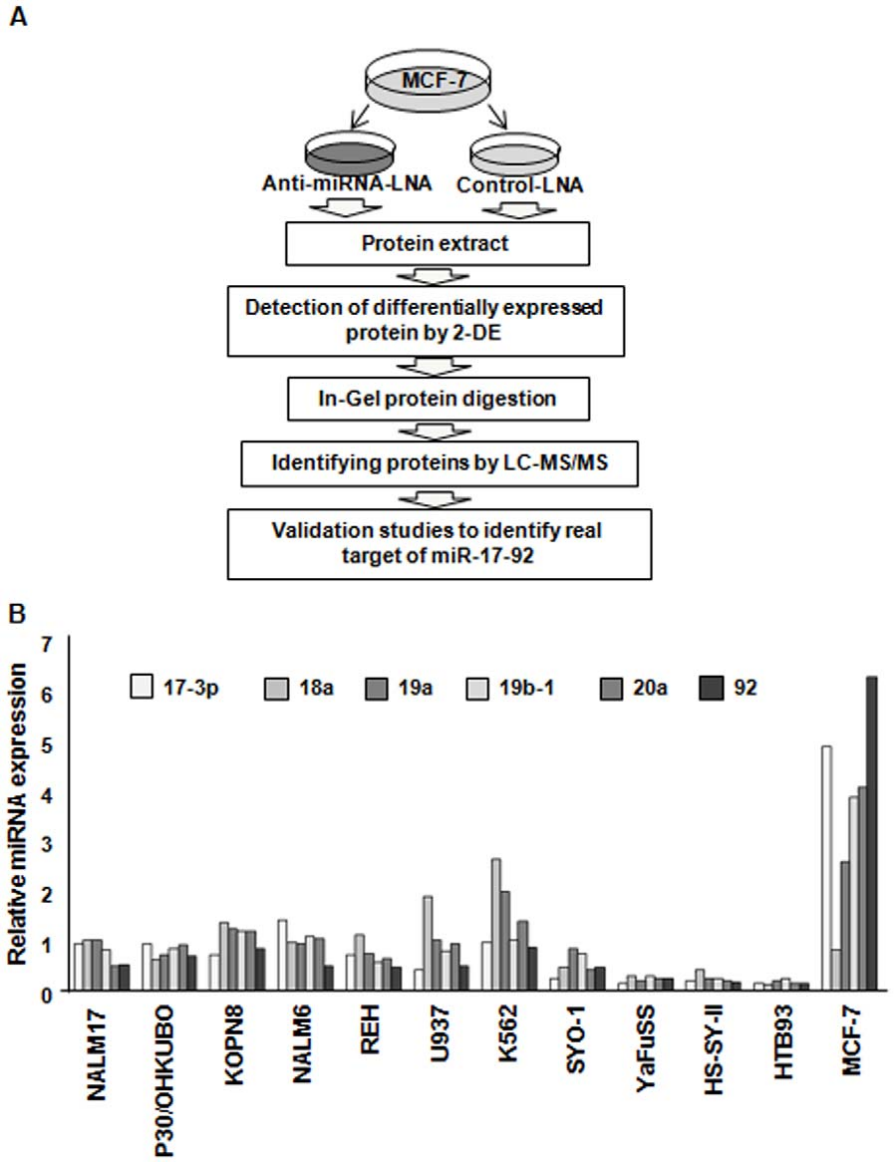
The strategy for identifying miR-17-92 targets. (A) The workflow for the identification of target proteins. MCF-7 cells were transfected with an anti-miRNA-LNA or control-LNA. Proteins were extracted 72 hours after transfection, and subjected to two-dimensional gel electrophoresis to detect differentially expressed proteins. Significantly overexpressed spots/proteins were digested with trypsin and identified by LC-MS/MS. (B) The expression of miR-17-92 in 12 human cancer cell lines. The expression level of each miRNA was normalized to that of an internal control, U6B RNA, in the same cell line, and was presented in comparison to the averaged value of the 12 cell lines for each miRNA.

To identify targets of miR-17-92, a 2-DE-based quantitative proteomic strategy was adopted (Fig. 1A). First, we screened 12 human breast cancer, lymphoma and synovial sarcoma cell lines to find cells that highly express miR-17-92. The expression of miR-17-3p, -18a, -19a, -19b-1, -20a and -92-1, which comprise the miR-17-92 cluster, in the 12 cell lines was analyzed using TaqMan real-time PCR. The relative expression level of each miRNA was calculated using the mean of the expression level in the 12 cell lines. We found that all of these miRNAs, except miR-18a, were expressed at a significantly highly level in MCF-7 breast cancer cells compared to the other cell lines (Fig. 1B). We hypothesized that knockdown of miR-17-92 would increase the protein products of its cognate target genes. In order to inhibit miR-17-92 expression, we transfected the MCF-7 cells with an anti-miRNA-LNAs against miR-19a, miR-20a, miR-92-1 or a negative control. Since the miR-17-92 cluster is categorized into four groups by alignment of their nucleotide sequences; the miR-17 family (miR-17-5p, miR-17-3p, miR-20a), the miR-18 family (miR-18a), the miR-19 family (miR-19a and miR-19b) and the miR-92 family (miR-92-1) [21], in this study, we focused on three representative miRNAs, miR-19a, miR-20a, and miR-92-1. MiR-18a was not selected because of its low level of expression in MCF-7 cells. A control LNA, targeting GFP, without any homology to mammalian miRNAs was used as a negative control.

**Figure 2:**
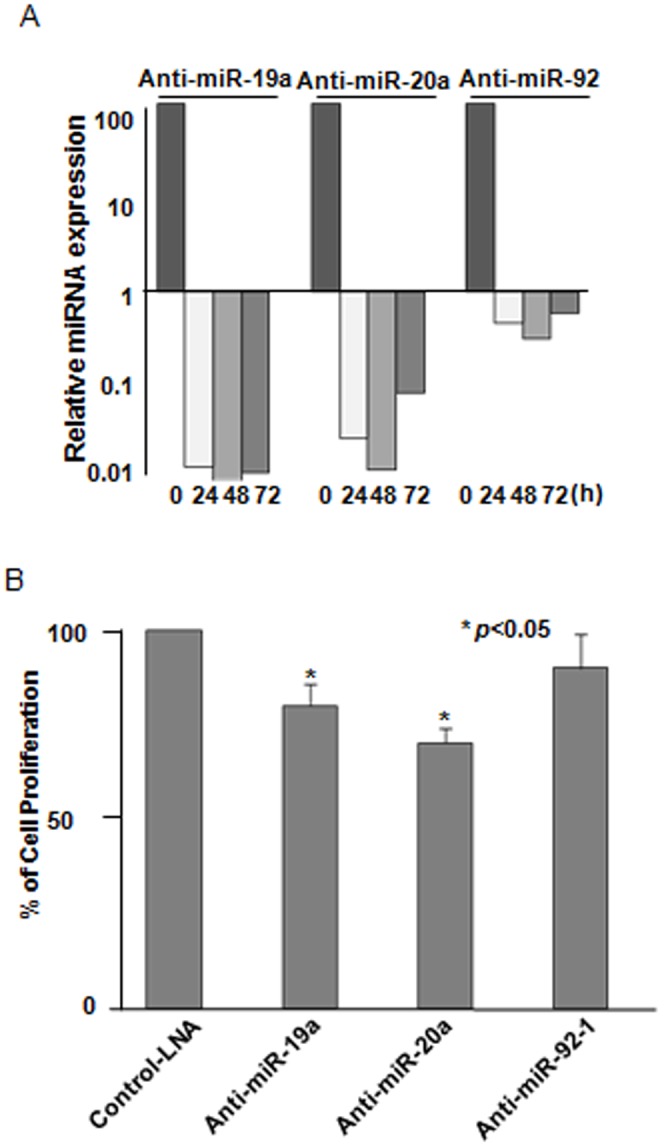
Effects of the anti-miR-LNA on MCF-7 cells. (A) The repression of miRNAs after the treatment of MCF-7 cells with the anti-miR-LNA. MCF-7 cells were transfected with an anti-miR-19a, anti-miR-20a, anti-miR-92-1 or control LNA. Significant repression of miR-19a (left), miR-20a (middle) and miR-92-1 expression (right) by the anti-miRNA-LNAs was observed from 24 to 72 hours after transfection. (B) Cell viability after the treatment of MCF-7 cells with the anti-miR-LNA. MCF-7cells were transfected with anti-miR-19a, anti-miR-20a, anti-miR-92 LNA and control LNA. Cell viability was determined using a WST-1 assay at 96 hours after transfection. The % cell proliferation was calculated by comparing the viability of cells treated with the anti-miR-LNAs compared to those treated with control LNAs.

To assess the knockdown efficiency of the anti-miRNA-LNAs, the expression of mature miR-19a, miR-20a and miR-92-1 were measured by TaqMan real-time PCR. The expression of miRNAs was dramatically decreased by the anti-miRNA-LNAs from 24 to 72 hours after transfection, although the inhibition efficiency was different between the miRNAs (Fig. 2A). The effect of the anti-miRNA-LNAs on cell viability was evaluated by a cell proliferation assay using WST-1. The cell growth rate of MCF-7 cells treated with an anti-miRNA-LNA compared to that of the cells treated with the control-LNA was 80.1% (miR-19a), 70.3% (miR-20a) and 90.2% (miR-92-1) at 96 hours after transfection (Fig. 2B).

### Detection and Identification of Candidate Targets of miR-19a, miR-20a and miR-92-1 by a Quantitative Proteomic Approach

The protein expression changes after knocking down miR-19a, miR-20a or miR-92-1 were analyzed by the quantitative proteomic approach outlined in Fig. 1A. Three independent 2-DE experiments were performed for each sample, and gels were subjected to fluorescent staining and/or silver staining. To identify and quantify the differentially expressed proteins that were specifically present in anti-miRNA-LNA-treated cells compared to control LNA-treated cells, the PDQuest software program (Bio-Rad) was used to evaluate the density of spots, and the results were shown as the percentage of the total optical density. A mean of 1455 valid spots per gel were obtained. Global differences between anti-miRNA-LNA-treated and control LNA-treated cells were not observed from the protein profiles of 2-DE gels, while 123 upregulated proteins were detected (53 proteins for miR-19a, 51 proteins for miR-20a, and 19 proteins for miR-92-1). The spots and the altered expression of these proteins are shown in Figs. S1 and S2, respectively. These spots were excised, digested with trypsin, and successfully identified by LC-MS/MS (Table S1).

**Figure 3:**
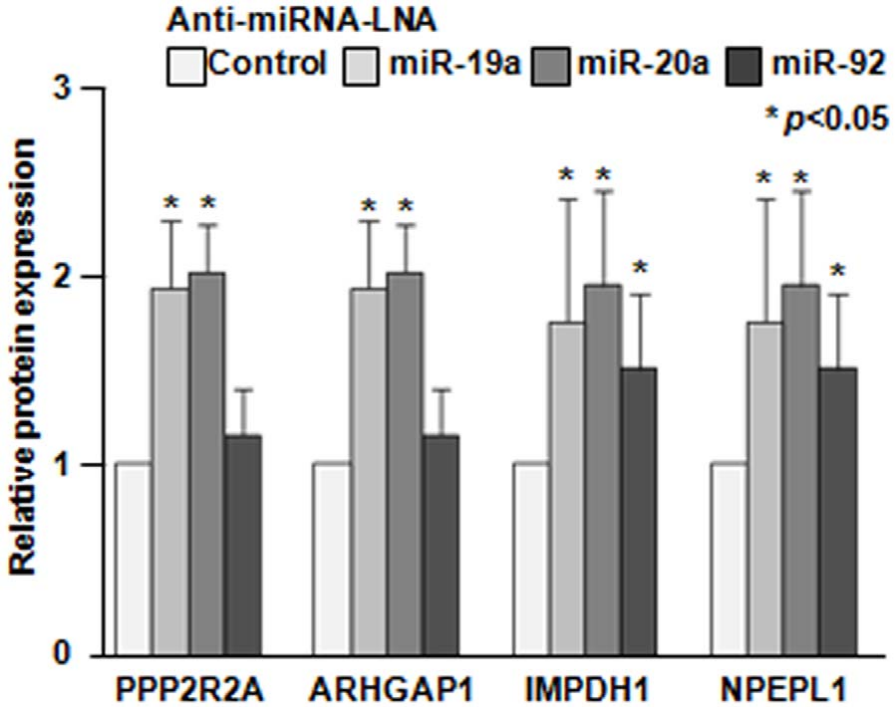
The relative expression levels of candidate proteins. The differentially expressed proteins from MCF-7 cells treated with anti-miRNA-LNAs compared to those treated with the control-LNA were identified and analyzed using the PDQuest Advanced Ver.8.0 software program (Bio-Rad), on the basis of their fluorescence intensity. *, *p*<0.05; **, *p*<0.01 using a two-tailed *t*-test (miRNA-LNA treated vs. control-LNA treated).

Among the identified upregulated proteins, 4 proteins were shown to have miR-19a or miR-20a binding sites on the 3′ UTR of their mRNAs. Fig. 3 shows the relative expression changes of the target proteins after treatment with the anti-miRNA-LNA. Representative MS/MS spectra of these proteins are shown in Fig. S3. Table 1 summarizes the candidate proteins and their symbols, UniProt accession numbers, MS/MS scores, number of spectra and peptides used for identification, amino acid coverage, molecular weight, theoretical pI and the miRNA binding sites as identified by LC-MS/MS and subsequent target prediction software programs. Serine/threonine-protein phosphatase 2A 55 kDa regulatory subunit B alpha isoform (PPP2R2A) was identified as a direct target candidate of miR-20a, and Rho GTPase-activating protein 1 (ARHGAP1), inosine-5′-monophosphate dehydrogenase 1 (IMPDH1) and probable aminopeptidase like-1 (NPEPL1) were identified as direct target candidates of miR-19a.

**Table 1:** Selection of candidate genes by computer-aided algorithms to search for miRNA target sites.

Genesymbol	UniProt accession #	MS/MS analysis							Target prediction		
		MS/MS score	Spectra	Peptides	AA^1^ coverage (%)		MW^2^ (kDa)	pI	PicTar	Target Scan	MiRanda
PPP2R2A	P63151	58.4	4	4		10	52	5.82	17	-	17, 20a
ARHGAP1	Q07960	41.35	3	3		6	50	5.85	17, 19a, 19b	19a	-
IMPDH1	P20839	29.81	3	3		6	55	6.43	19a, 19b	-	19a
NPEPL1	Q8NDH3	18.26	1	1		2	56	6.41	19a, 19b	-	-

1 AA: amino acid.

2 MW: molecular weight.

### Verification of the Direct Targets of miR-19a and miR-20a

**Figure 4:**
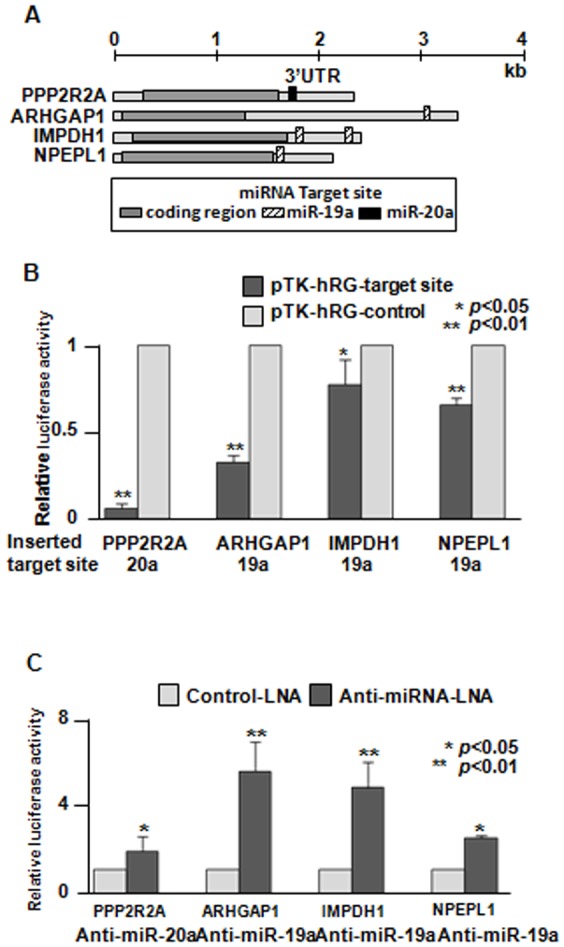
Verification of the results of the proteomic analysis data by luciferase assays. (A) Overview of target mRNAs. The miRNA target sites were identified in the 3′ UTR of mRNAs using the Pictar, TargetScan or MiRanda software program. The 3′ UTR fragments containing the miRNA target regions were synthesized as oligonucleotides and cloned into the 3′ UTR of the Renilla luciferase of the pTK-hRG plasmid. (B) The luciferase activity of cells treated with constructs containing the miR-17-92 target sites was compared to that of cells treated with the control vectors, which had the same length of each target site, but in the reverse orientation. The data are shown as the means + SD. *, *p*<0.05; **, *p*<0.01 using a one-way ANOVA followed by Scheffe’s F-test (pTK-hRG-target site vs. pTK-hRG-control). (C) An anti-miRNA-LNA or control-LNA was co-transfected with the pTK-hRG-target site plasmids. The luciferase activity of anti-miRNA-LNA-treated group was compared to that of the control-LNA treated group. The data are shown as the means + SD. *, *p*<0.05; **, *p*<0.01 using a one-way ANOVA followed by Scheffe’s F-test (anti-miRNA-LNA vs. control-LNA).

To confirm that miR-19a or miR-20a directly regulates the expression of these candidate targets, we performed luciferase assays. First, the nucleotide sequences of the miRNA binding sites on the 3′ UTR of these target mRNAs were obtained from the GenBank database (depicted in Fig. 4A). The 3′ UTR of these targets were individually cloned downstream of the luciferase ORF in the pTK-hRG vector (Fig. S4). Next, each construct was transfected into MCF-7 cells. The luciferase activity significantly decreased in all of the targets (Fig. 4B).

In order to validate the activity by another luciferase assay, each construct was co-transfected into MCF-7 cells with anti-miRNA-LNA or control LNA. The luciferase activity was significantly increased for all candidate targets after miRNA inhibition (Fig. 4C), which was in contrast to the previous luciferase assay.

**Figure 5:**
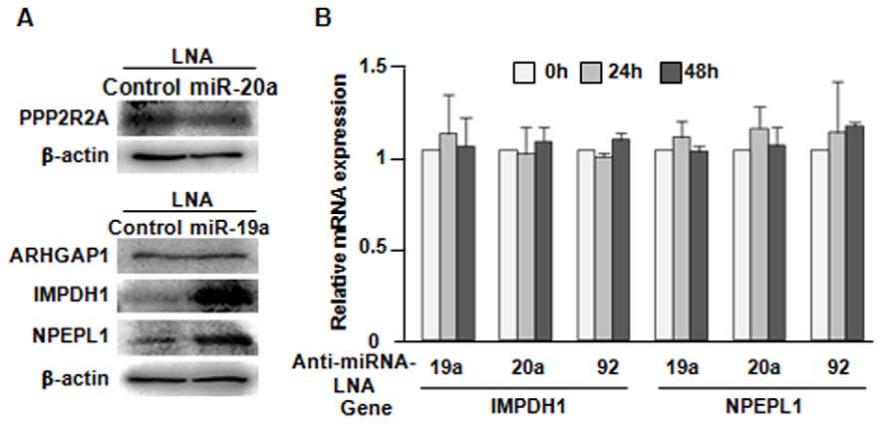
Verification of the candidate targets. (A) Verification of the candidate proteins by a Western blot analysis. The protein extracts from MCF-7 cells treated with the anti-miRNA-LNA or control-LNA were evaluated. (B) The effect of the miRNAs on the levels of IMPDH1 and NPEPL1 mRNAs. The expression of the mRNAs in the anti-miRNA-LNA-treated groups was compared to that in the control LNA-treated group by quantitative real-time PCR. The miRNA inhibition by the anti-miRNA-LNA had no significant effect on either the IMPDH1 or NPEPL1 mRNA levels from 24 to 72 hours after transfection. The data are shown as the means + SD.

The protein expression changes of candidate targets after treatment with an anti-miRNA-LNA were examined by a Western blot analysis in MCF-7 cells in order to confirm the data obtained from the luciferase assay. The expression levels of IMPDH1 and NPEPL1 were both increased after treatment with anti-miR-19a, while the expression levels of PPP2R2A and ARHGAP1 did not change (Fig. 5A).

Quantitative real-time PCR was performed as the last validation step to examine whether there were any changes in the expression of *IMPDH1* and *NPEPL1* at the mRNA level. As expected, the expression levels of *IMPDH1* and *NPEPL1* were not significantly changed by anti-miR-19a-LNA at the mRNA level (Fig. 5B), while the miR-19a expression was decreased following the anti-miR-19a-LNA treatment (Fig. 2A). Taken together, our results indicate that miR-19a directly affects the post-transcriptional regulation of the *IMPDH1* and *NPEPL1* genes.

**Figure 6:**
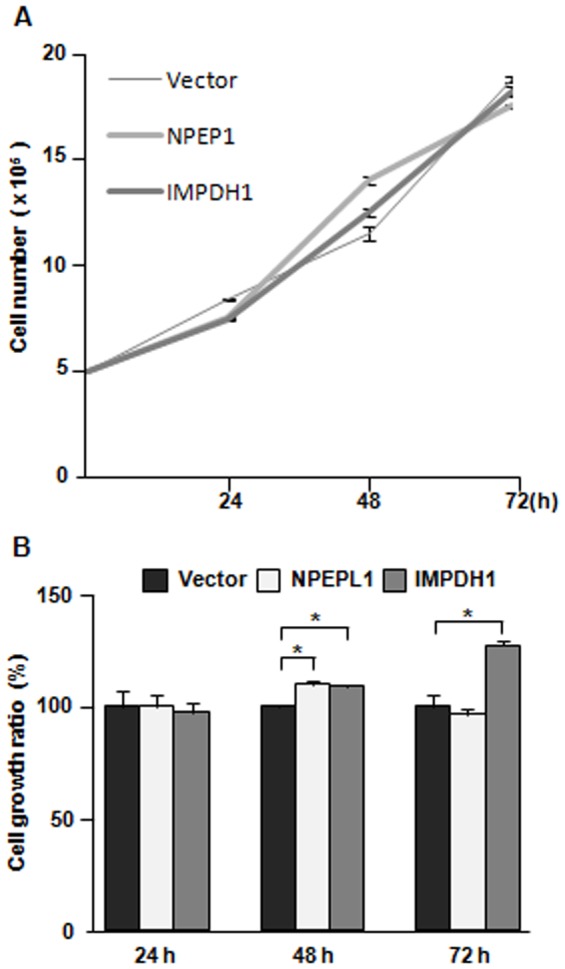
Effects of exogenous expression of target genes on cell growth of MCF-7. MCF-7 cells were transfected with the *IMPDH1*, *NPEPL1* cDNA expression vector or a control vector and then counted and split into multi-well plates at 24 hours after transfection. Cell viability was measured with the Trypan Blue excision test (A) and a WST-1 cell proliferation assay (B) at 24, 48 and 72 hours after the split. For the WST-1 assay, the cell growth ratio (%) was calculated by comparing the viability of cells treated with the target genes compared with that of the cells treated with the control vector.

In order to examine the effects of the *IMPDH1* and *NPEPL1* genes on growth suppression of breast cancer cells, the GFP expression vectors bearing the *IMPDH1* or *NPEPL1* gene were transfected into MCF-7 cells by electroporation. By observing GFP fluorescence at 24 hours after electroporation, it was confirmed that the transfection efficiency between these transfectants was almost equivalent (approximately 80%). These cells were counted and split onto 96- and 6-well plates, and the cell growth was measured using the cell proliferation reagent WST-1 and Trypan Blue exclusion test at 24, 48 and 72 hours after the split. If these genes are associated with tumorigenesis under the control of miR-19a, then the cells with the exogenous expression of these genes will show a decreased cell growth. However, cells transfected with the *IMPDH1* or *NPEPL1* gene did not show a decreased growth in comparison to the cells transfected with a control vector (Fig. 6).

## Discussion

Recently, the association between the abnormal expression of miRNAs and tumorigenesis was reported. However, the molecular mechanisms by which miRNAs can modulate tumor growth or metastases remain unknown. In particular, the activities and importance of the miR-17-92 cluster are largely unknown in breast cancer. One of the reasons may be our limited knowledge of miRNA targets. The most characteristic feature of miRNAs is the fact that they regulate gene expression by translational repression rather than mRNA degradation [1]. Considering this characteristic of miRNAs, we hypothesized that a comprehensive proteomic approach would be one of the most rational ways to directly detect miRNA targets. Therefore, we employed a miRNA knockdown system using antisense-miRNA-LNAs to identify the differentially expressed proteins on 2-DE gels. This system was expected to provide improved results compared to the previous approaches using sense-miRNA transfection (expression vectors) systems, which did not provide sufficient data to identify the differentially expressed target proteins (data not shown).

The results of our proteomic analysis were compared to the predicted results for potential targets of miR-19a, -20a and -92-1 using the Pictar (http://www.pictar.org/), TargetScan (http://targetscan.org/), and MiRanda (http://www.microrna.org/microrna/home.do) software programs. Although only a few putative targets were identified by these three prediction programs, there is a possibility that false-positives or negatives were generated from the search using the computational prediction systems based on miRNA seed regions. Thus, we performed additional experimental analyses to increase the accuracy of the prediction programs and the likelihood of detecting genuine direct targets of miRNAs. Finally, the candidate proteins which were identified by the 2-DE analysis were validated by luciferase assays and a Western blot analysis to eliminate the possibility of false positive results.

Four novel candidate targets (PPP2R2A, ARHGAP1, IMPDH1 and NPEPL1) were identified by our proteomic approach. Their miRNA binding sites were shown to be important for their translation, as indicated by the luciferase activity assays. The luciferase activity of the plasmids with each target site was decreased (Fig. 4B), and increased luciferase activity was observed in the presence of the specific anti-miRNA-LNA (Fig. 4C). The IMPDH1 and NPEPL1 proteins were confirmed to be potential direct targets of miR-19a by a Western blot analysis (Fig. 5A). As miR-19a was expressed at a significantly highly level in MCF-7 cells, it is considered that the IMPDH1 and NPEPL1 proteins might function as tumor suppressors if they are associated with tumorigenesis under the control of miR-19a. However, we could not find any growth suppression effects of these genes on MCF-7 cells (Fig. 6). In the present study, we could not validate the impact of miRNAs in this cluster on any tumor suppressor proteins, although we tried to identify some genes that have tumor suppressor activity that are direct targets of miR-17-92.

IMPDH1 (Inosine-5-prime-monophosphate dehydrogenase) catalyzes the rate-limiting step in the *de novo* synthesis of guanine nucleotides, *i.e*., the formation of xanthine monophosphate from inosine monophosphate [25]. IMPDH1 is a ubiquitously expressed enzyme, functioning as a homotetramer, and it may play an important role in cyclic nucleotide metabolism within photoreceptors. Mutations and decreased expression of the *IMPDH1* gene are responsible for the disease phenotype of autosomal dominant retinitis pigmentosa [26], [27]. The bulk of GTP within photoreceptors is generated by IMPDH1, and dysfunction of this enzyme might give rise to neurodegeneration [28] **.** Decreased expression of the IMPDH enzyme by aberrant overexpression of miR-19a might play a role in some cases of this disease. In general, guanine nucleotides are synthesized through the *de novo* synthesis pathway in T- and B-lymphocytes, while there are two synthetic pathways, the *de novo* pathway and a salvage pathway, in epithelial cells. Some IMPDH inhibitors, e.g. mycophenolic acid and mycophenolate mofetil, are known to have immunosuppressive activity through the *de novo* synthesis of guanine nucleotides in lymphocytes. IMPDH inhibitors are also known to have antiviral activity and tumor suppressor activity. Therefore, the inhibition of IMPDH1 by miR-19a may represent a potential strategy for antitumor-, antiviral- and immunosuppressive therapies.

The *NPEPL1* (probable aminopeptidase-like 1) gene is located on chromosome 20q13.32, and has been deposited in the NCBI database (NCBI Gene ID, 79716). However, no information about the function of the protein has been reported so far.

The miR-17-92 cluster was identified as an oncogenic cluster of miRNAs with multifaceted functions in cell survival, proliferation and differentiation [19], [24], [29]. This cluster is known to be upregulated in lung cancer [24] and B-cell lymphomas [19], and downregulated in senescent cells [30]. The suppressor of cytokine signaling-1 (*SOCS-1*) gene [31] has been reported as a target of miR-19a, and retinoblastoma-like protein 2 (*RBL2*) [32], hypoxia-inducible factor 1-alpha (H*IF1A*) [33] and Ras-related protein Rab-14 (*RAB14*) [34] have been identified as direct targets of miR-17-92. Recently, HMG box-containing protein 1 (*HBP1*) [35] and zinc-finger and BTB domain containing 4 (*ZBTB4*) [36] were reported to be targets of miR-17-92 and to be correlated with the prognosis in breast cancer. Recently, miR-19a was identified as a key molecule responsible for the oncogenic activity of the cluster, and was shown to reduce the tumor suppressor PTEN level, and consequently activate the AKT/mTOR (mammalian target of rapamycin) pathway [37], [38].

Although miRNAs are largely known to repressively regulate protein expression, it has been reported that some miRNAs can also upregulate translation [39]. In this study, we focused on the repressive gene regulation of the miRNAs as a result of their binding to the 3′UTR of target genes, and identified only proteins that were downregulated by miR-17-92, while many upregulated proteins were also detected by the 2-DE analysis. Further experiments will be needed to determine whether any of these upregulated genes are targets of miR-17-92, and how some miRNAs are able to upregulate translation. Furthermore, a previous study demonstrated that the suppression of miR-17-92 induces complete growth arrest in anaplastic thyroid cancer cells [40], while the overexpression of one of its members, miR-20a, induces senescence in mouse embryonic fibroblasts [41], indicating that special attention needs to be paid to the possible cell type-specific responses to miR-17-92.

In conclusion, we found that miR-17-92 is overexpressed in MCF-7 breast cancer cells, and performed direct target profiling of miR-17-92 in these breast cancer cells. We identified 123 genes that were candidate targets of miR-19a, miR-20a or miR-92-1 using a quantitative proteomic approach, and performed subsequent validation studies on several of these candidates. Among these candidate targets, *IMPDH1* and *NPEPL1* were identified as novel direct targets of miR-19a in the MCF-7 breast cancer cells. Overall, our validation studies based on luciferase assays, Western blot analyses and quantitative real-time PCR clearly showed that miR-19a regulates the expression of the *IMPDH1 and NPEPL1* genes at the translational level, without affecting their mRNA expression levels. This is accomplished through not only a single seed region, but also likely through multiple seed regions. Further investigations will be required to clarify the proteins and mechanism(s) of tumorigenesis mediated by miR-17-92 in breast cancer by using the methods for identifying direct miRNA targets such as proteomic approaches.

## Materials and Methods

### Cell Culture

Synovial sarcoma cell lines SYO-1, YaFuSS, and HS-SY-II were provided by Dr. A. Kawai (National Cancer Center, Tokyo, Japan) [42], Dr. J. Toguchida (Institute for Frontier Medical Sciences, Kyoto University, Japan) [43], and Dr. H. Sonobe (National Fukuyama Hospital, Hiroshima, Japan) [44], respectively. Synovial sarcoma cell line HTB-93, breast cancer cell line MCF-7, lymphoma cell line U937, and chronic myelogenous leukemia cell line K562 were purchased from American Type Culture Collection. Hematopoietic cell lines NALM-7, p30/OHKUBO, KOPN-8, NALM-6 and REH were supplied by Hayashibara Inc. (Okayama, Japan). These cell lines were grown in Dulbecco’s modified Eagle’s medium or RPMI-1640 (Invitrogen, Carlsbad, CA, USA) supplemented with 10% fetal bovine serum (Invitrogen), 100 units/ml of penicillin G and 100 µg/ml of streptomycin (Meiji Seika, Tokyo, Japan). All cells were incubated at 37°C in a humidified atmosphere containing 5% CO_2_.

The knockdown of miR-19a, miR-20a and miR-92 was performed using anti-miRNA LNAs (Gene Design, Inc, Osaka, Japan). The cells were plated in 92 mm culture dishes or 24 well plates and then transfected with anti-miRNA LNA oligonucleotides (30 nM) using lipofectamine 2000 (Invitrogen). A control LNA oligonucleotide against GFP was included in a parallel experiment. The cells were subjected to RNA extraction or luciferase assay 48 hours after transfection, and protein extraction 72 hours after transfection. Cell viability was determined using a WST-1 assay (Roche Applied Science, Mannheim, Germany) at 96 hours after transfection, according to the manufacturer’s instructions.

### Quantitative Real-time PCR

Total RNA was extracted using ISOGENE (Nippon Gene, Tokyo, Japan) according to standard guanidium-phenol-chloroform extraction procedures. The quantitative real-time PCR analysis of miRNAs contained within the miR-17-92 cluster was performed with a TaqMan MicroRNA Reverse Transcription Kit, TaqMan Universal PCR Master Mix and TaqMan MicroRNA Assay (Applied Biosystems, Foster City, CA, USA). Total RNA (10 ng) was reverse transcribed in a total volume of 15 µL using a TaqMan MicroRNA Reverse Transcription kit. Aliquots of each RT reaction were amplified by PCR in a 20 µL total volume containing 10 µL of the TaqMan 2X Universal PCR Master Mix. The PCR was performed on a 7300 Fast Real-Time PCR System with an initial incubation at 95°C for 15 s and 60°C for 60 s. Each PCR reaction was performed in triplicate a minimum of three times. The expression level, i.e. cycle threshold (CT) value, of each miRNA was normalized to the CT value of a small nuclear RNA, U6B, which was co-amplified as an endogenous control. The ΔCT was calculated as the difference in the CT values between the tested miRNA and the internal control in one sample. The comparisons of miRNA expression levels were conducted using the ΔΔCT method, where the ΔΔCT was the difference in the ΔCT values between two samples and 2^−ΔΔCT^ represents the fold change in miRNA expression. After the ΔCT of the miRNA in human cancer cell lines was averaged, a comparison of the miRNA expression level in the MCF-7 cells to the average value was made using the ΔΔCT method, as shown in Fig. 1B.

Quantitative real-time PCR for the candidate target genes, *NPEPL1* and *IMPDH1,* was performed with ReverTra Ace First Standard cDNA Synthesis Kit (TOYOBO, Osaka, Japan) and POWER SYBR Green PCR Master Mix (Applied Biosystems). The primer sequences were as follows; *NPEPL1* forward, 5′-CTC TTC ATC GCC TCA CAC ATC-3′ and reverse, 5′-TCA CAC AAG CCT GCG TCT CTT-3′; *IMPDH1* forward, 5′-CCA TGA TGT ACT CAG GAG AGC-3′ and reverse, 5′-ACC CGT AGT GCA AAT CTG TGG-3′. The CT values were normalized to the CT value of the *GAPDH* gene in the same sample. The expression levels of the target mRNA were also measured using the ΔΔCT method. The expression changes of miR-17-92 or the candidate target genes after treatment with the anti-miRNA LNA were also measured.

### Protein Extraction and Two-dimensional Electrophoresis

The cells in exponential growth phase were washed with PBS and harvested by mechanical scraping. Cells were centrifuged, and the cell pellets were solubilized in lysis buffer consisting of 5 M urea, 2 M thiourea, 2% CHAPS, 2% SB3-10, 1% DTT and a protease inhibitor cocktail (Sigma-Aldrich, St. Louis, MO, USA). After 3 freeze-thaw cycles, the cells were sonically disrupted for 30 s, and ultracentrifuged at 75,000× g for 30 min at 10°C using an Optima™ TLF Ultracentrifuge (Beckman Coulter, Brea, CA, USA). The supernatant was transferred to a new tube and treated with a ReadyPrep 2D Cleanup Kit (Bio-Rad, Hercules, CA, USA) to remove ions, DNA, RNA, etc. The protein concentration was determined using the RC-DC Protein Assay (Bio-Rad) according to the two-wash standard protocol. Pharmalyte 3–10 for isoelectric focusing was formulated to increase the resolution at the basic end of a flatbed isoelectric focusing gel.

Each of the samples was diluted in rehydration buffer containing 5 M urea, 2 M thiourea, 2% CHAPS, 3% SB3-10, 1% DTT and 0.2% Bio-Lyte® 3/10 ampholyte (Bio-Rad), to give a final sample volume of 300 µL containing 60 µg of total protein, thereby ensuring that a consistent amount of protein was applied to each strip. Samples were applied by rehydration for 15 hours on a separate nonlinear immobilized pH gradient DryStrip (17 cm, pH 3–10, Bio-Rad), and focused in a Bio-Rad Protean IEF cell using the following voltage program: 250 V for 40 m, 10,000 V for 4 hours, and a third step of a total 70,000 V-h, and then the current was maintained at 500 V.

The focused strips were then equilibrated in buffer I (6 M urea, 2% SDS, 0.375 M Tris-HCl pH8.8, 20% Glycerol, 2% DTT) for 30 min and then buffer II (6 M urea, 2% SDS, 0.375 M Tris-HCl (pH 8.8), 20% glycerol, 2.5% iodoacetamide) for 15 min with gentle shaking. The second dimensional separation was performed on 12% SDS-polyacrylamide gels using a PROTEAN II Cell (Bio-Rad) at 20°C and a 40 mA/gel constant amperage for 4 hours. The gels were then stained with SYPRO Ruby (Invitrogen) or silver stain according to the manufacturer’s protocol.

### Image Analysis of 2-DE Gels

The images of SYPRO Ruby-stained gels were obtained using an image analyzer FLA-3000 (Fujifilm, Tokyo, Japan) and were analyzed using the PDQuest Advanced Version 8.0 software (Bio-Rad), which included background subtraction, spot detection and volume normalization. The intensity of each spot was quantified by calculation of the spot volume after normalization according to the local regression model method. The intensities of matched spots were compared, and a threshold was set at 1.5-fold to screen for differences. Visual inspection confirmed the differences indicated by the PDQuest software package.

### Protein Identification

Protein spots of interest were manually excised from silver stained gels, and then were destained and dried. In-gel trypsin digestion using a Protein In-Gel Tryptic Digestion Kit (Agilent Technologies, Santa Clara, CA, USA) was done at 30°C overnight. The peptide digests obtained were analyzed with a nano-flow liquid chromatography-ion trap-tandem mass spectrometer (nLC-IT-MS/MS, Agilent 1100 LC/MSD Trap XCT Ultra, Agilent Technologies) in a fully automated manner.

The identification of proteins was performed using the Spectrum Mill MS Proteomics Workbench platform (version A.03.02, Agilent Technologies) according to the workflow of Spectrum Mill. The identification parameters were set as follows: database, NCBInr; enzyme, trypsin; monoisotopic masses were used; precursor mass tolerance (peptide tolerance), +/−2.5 Da; product mass tolerance (MS/MS tolerance), +/−0.8; the fixed modification was selected as carbamidomethylation (cysteine); the variable modification was selected as oxidation (methionine), two missed cleavages with trypsin were allowed, and the instrument setting was specified as “ESI ion trap”. The probability scores calculated by the software were used as a criterion for correct identification.

### Luciferase Reporter Assay

The 3′ UTR fragments containing a possible miRNA binding region in the candidate genes were synthesized as oligonucleotides for both strands which could produce *Xba*I cohesive ends after annealing. Otherwise, the 3′ UTR fragments were amplified by PCR with human cDNA, using PCR primer pairs with an *Xba*I restriction enzyme site. The resulting double strands were cloned into the pTK-hRG vector at the 3′ UTR *Xba*I site of Renilla luciferase, which was a phRG-B vector (Promega, Madison, WI, USA) with the herpes thimidine kinase promoter. The inserted fragments were sequenced, and their orientation and fragment number were confirmed. The target sequences cloned into the vectors are shown in Fig S4. MCF-7 cells were plated on 48-well plates (5×10^4^ cells per well). The pTK-hRG constructs (180 ng) were co-transfected with the firefly luciferase reporter plasmid, pOA-SRα-luciferase (20 ng), as an internal control using Lipofectamine 2000 (Invitrogen). The pTK-hRG constructs with the reverse orientation were used as controls for those with the forward orientation. Otherwise, the pTK-hRG constructs with the forward orientation were co-transfected with each anti-miRNA-LNA or control-LNA (100 nM) under the same conditions. An anti-GFP-LNA was used as a control for transfection with the anti-miRNA-LNA. The luciferase activity was measured 48 hours after transfection using a dual luciferase reporter assay system (Promega) on a Labosystems Luminoskan RT instrument (Thermo Scientific). The relative luciferase activity was calculated by normalizing the firefly luminescence to the renilla luminescence.

### Western Blotting Analysis

The cells were lysed in the same buffer used for the 2-DE analysis. A total of 20 µg of whole protein lysates were combined with gel loading buffer, heated to 95°C for 10 min, and then separated on 12% SDS-polyacryl-amide gels and electrotransferred to polyvinylidene difluoride membranes (Invitrogen). Membranes were blocked overnight at 4°C in 3% BSA/PBS, and then incubated for 4 hours at room temperature with the following antibodies: 1∶1000 rabbit polyclonal anti-PPP2R2A (ab18136, Abcam, Cambridge, UK), 1∶250 rabbit polyclonal anti-ARHGAP1 (ab72127, Abcam, Cambridge, UK), 1∶200 mouse monoclonal anti-IMPDH1 (H00003614-M01, Abnova, Taipei City, Taiwan) and 1∶200 mouse monoclonal anti-NPEPL1 (sc-100556, Santa Cruz Biotechnology). β-actin was used as a loading control and was detected by a 1∶1000 mouse monoclonal anti-β-actin antibody (A5316, Sigma, Saint Louis, USA). After washing with PBS/0.05% Tween-20, the membranes were incubated with alkaline phosphatase-conjugated secondary antibodies in PBS. Signals were measured by an enhanced chemiluminescence detection system using a VECTASTAIN ABC-AmP Chemiluminescent Detection Kit (VECTOR LABORATORIES, Inc) and visualized using FLA-3000 (Fujifilm).

### Exogenous Expression of Target Genes in MCF-7 Cells

The *IMPDH1* and *NPEPL1* cDNAs were amplified using PCR with human normal cDNA and primers (IMPDH1 forward, 5′-CTC GAG ACC ATG GAT CGC CTT CGC AGG GCT and IMPDH1 reverse, 5′-CTC GAG TCA GTA CAG CCG CTT TTC GTA GA; NPEPL1 forward, 5′-GAA TTC GCC ACC ATG GCG AAC GTG GGG CTG CAG TTC and NPEPL1 reverse, 5′-GGA TCC TCA CAC AAG CCT GCG TCT CTT GGA) and cloned into pBluescript. The nucleotide sequences were confirmed with DNA sequencing and the cDNA fragments were digested with restriction enzymes (*Xho*I for the *IMPDH1* and *Eco*RI and *Bam*HI for the *NPEPL1*) and cloned into pIRES2-EGFP to generate pIMPDH1-IRES-EGFP and pNPEPL1-IRES-EGFP plasmids.

The pIMPDH1-IRES-EGFP, pNPEPL1-IRES-EGFP and control pEGFP plasmids were transfected to MCF-7 cells using the Neon Transfection system (Invitrogen, 1100 V, 30 ms, 2 pulses). After 24 hours, it was confirmed by observing the GFP fluorescence that the transfection efficiency between these cells was almost equal (approximately 80%), and the cells were counted and split into 96-well plates (1×10^3^ cells/well) and 6-well plates (5×10^5^ cells/well). At 24, 48 and 72 hours after the split, cell viability was measured using 6-well plates and the Trypan Blue excision test and using 96-well plates and a WST-1 assay (Roche Applied Science), according to the manufacturer’s instructions. For the WST-1 assay, the cell growth ratio (%) was calculated by comparing the viability of cells treated with the *IMPDH1* or *NPEPL1* genes compared to that of cells treated with a control vector.

### Statistical Analysis

All experiments were repeated independently a minimum of three times, and the results are expressed as the mean values + SD. The relative expression of miRNAs, mRNAs or proteins was analyzed by paired *t*-tests. The other results were assessed by a one-way ANOVA followed by Scheffe’s F-test. A value of *p*<0.05 was considered to indicate statistical significance.

## Supporting Information

Figure S1 **A representative gel image of MCF-7 cells treated with the anti-miRNA-LNA after fluorescent staining.** Protein spots indicated with red circles were positive candidates for regulation by miR-17–92. The molecular weight marker is indicated at left.
(TIF)

Figure S2 **A representative enlarged gel image and spots of candidate targets.** The protein spots indicated by arrowheads are target spots on the gels.
(TIF)

Figure S3 **The MS/MS spectra used to identify candidate proteins.** The panels show the MS/MS spectra of representative peptides; (A) Serine/threonine-protein phosphatase 2A 55 kDa regulatory subunit B alpha isoform (PPP2R2A), (B) Rho GTPase-activating protein 1 (ARHGAP1), (C) Inosine-5′-monophosphate dehydrogenase 1 (IMPDH1), (D) Probable aminopeptidase NPEPL1 (NPEPL1).
(TIF)

Figure S4 **Construction of the luciferase vectors.** The miR-17–92 target sites of the candidate genes were cloned downstream of the luciferase ORF at the *Xba*I restriction site of the pTK-hRG vector. The miRNA target sites are shown as nucleotide sequences (left) and as boxes (right). Sense (upper) and antisense (lower) strands of complementary sequences indicate the miRNA target site of mRNA 3′ UTR and the corresponding miRNA sequences, respectively. Seven nucleotides (red) on miRNAs show the seed sequences for binding with mRNA.
(TIF)

Table S1 **Candidate targets of the miR-17–92 cluster identified by LC-MS/MS.**
(TIF)
