# Supplementary figures and images for: Novel Direct Targets of miR-19a Identified in Breast Cancer Cells by a Quantitative Proteomic Approach
Source: PLoS One. 2012 Aug 30;7(8):e44095. doi: 10.1371/journal.pone.0044095 (PMC3431339; doi:10.1371/journal.pone.0044095)

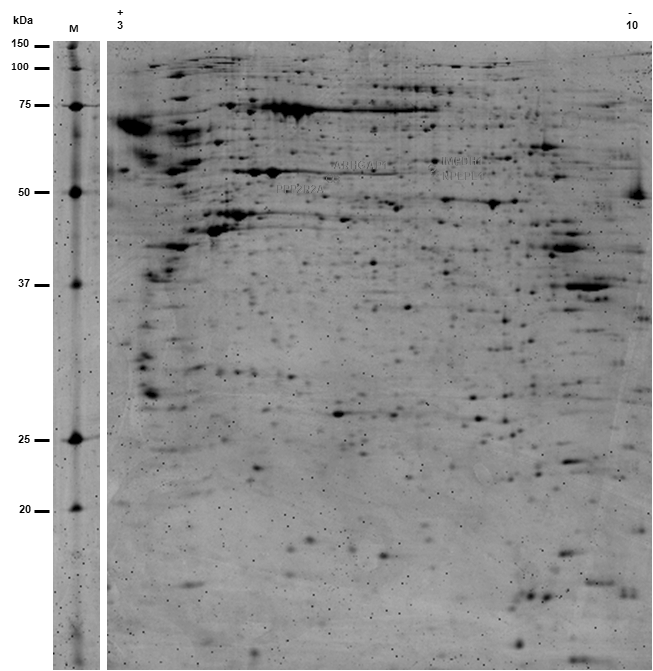

Supplement: Figure S1 — A representative gel image of MCF-7 cells treated with the anti-miRNA-LNA after fluorescent staining. Protein spots indicated with red circles were positive candidates for regulation by miR-17–92. The molecular weight marker is indicated at left. (TIF) [file pone.0044095.s001.tif]

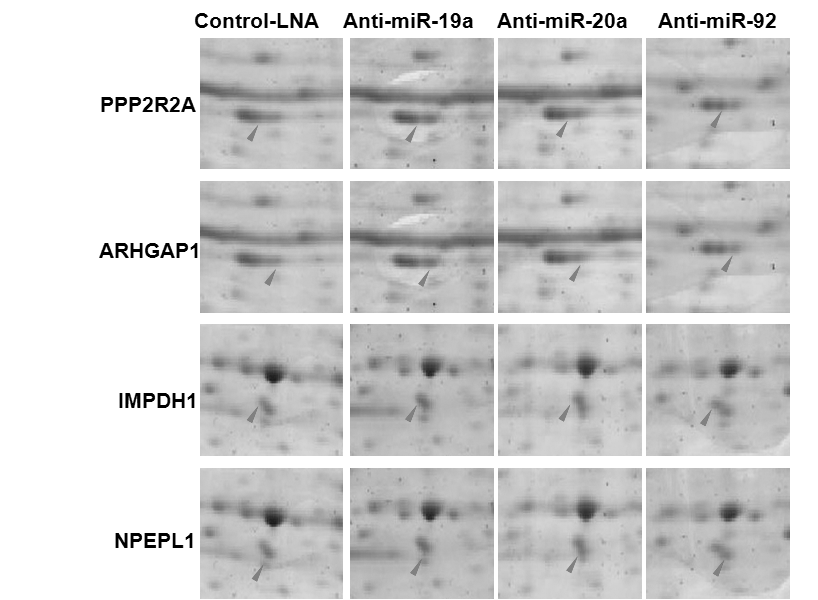

Supplement: Figure S2 — A representative enlarged gel image and spots of candidate targets. The protein spots indicated by arrowheads are target spots on the gels. (TIF) [file pone.0044095.s002.tif]

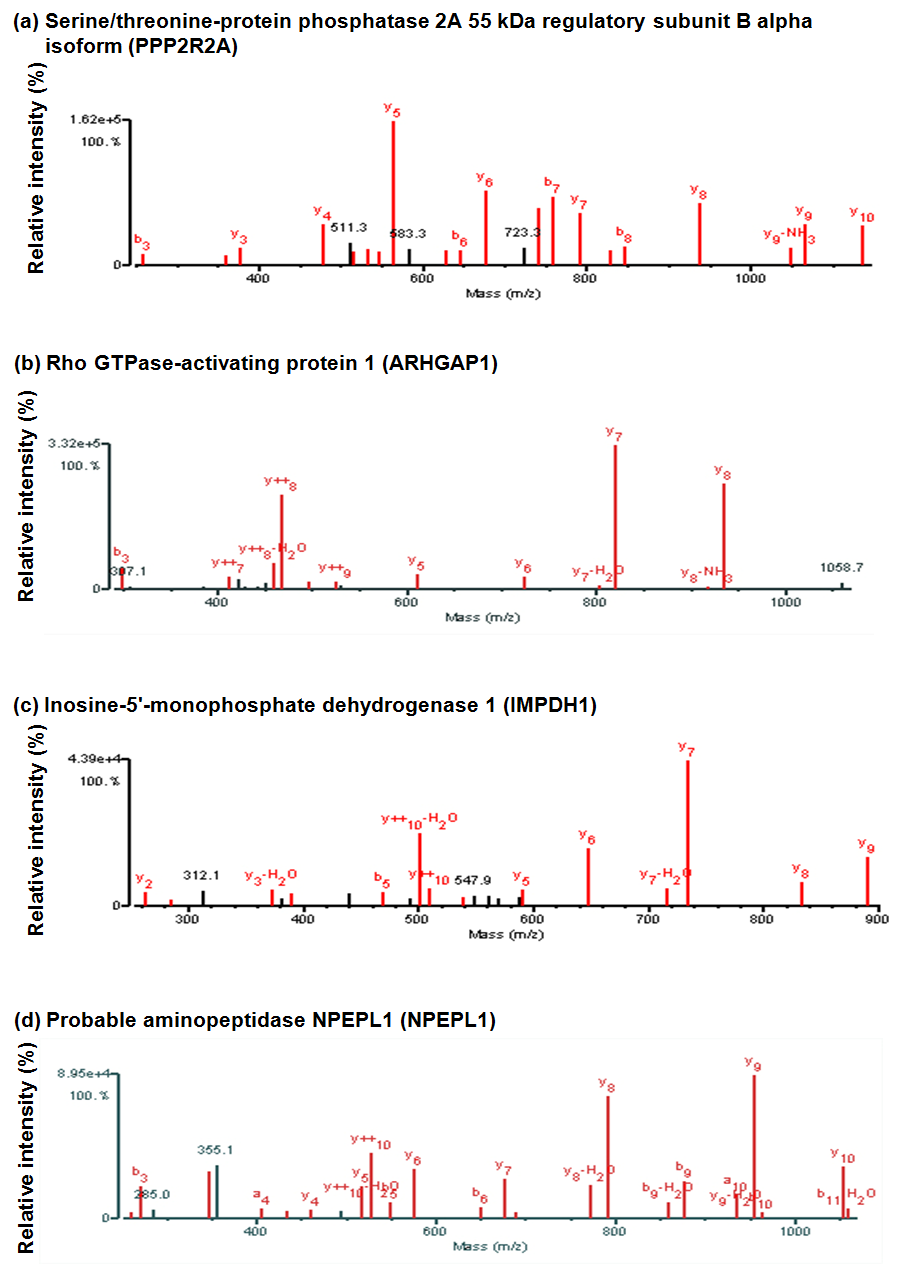

Supplement: Figure S3 — The MS/MS spectra used to identify candidate proteins. The panels show the MS/MS spectra of representative peptides; (A) Serine/threonine-protein phosphatase 2A 55 kDa regulatory subunit B alpha isoform (PPP2R2A), (B) Rho GTPase-activating protein 1 (ARHGAP1), (C) Inosine-5′-monophosphate dehydrogenase 1 (IMPDH1), (D) Probable aminopeptidase NPEPL1 (NPEPL1). (TIF) [file pone.0044095.s003.tif]

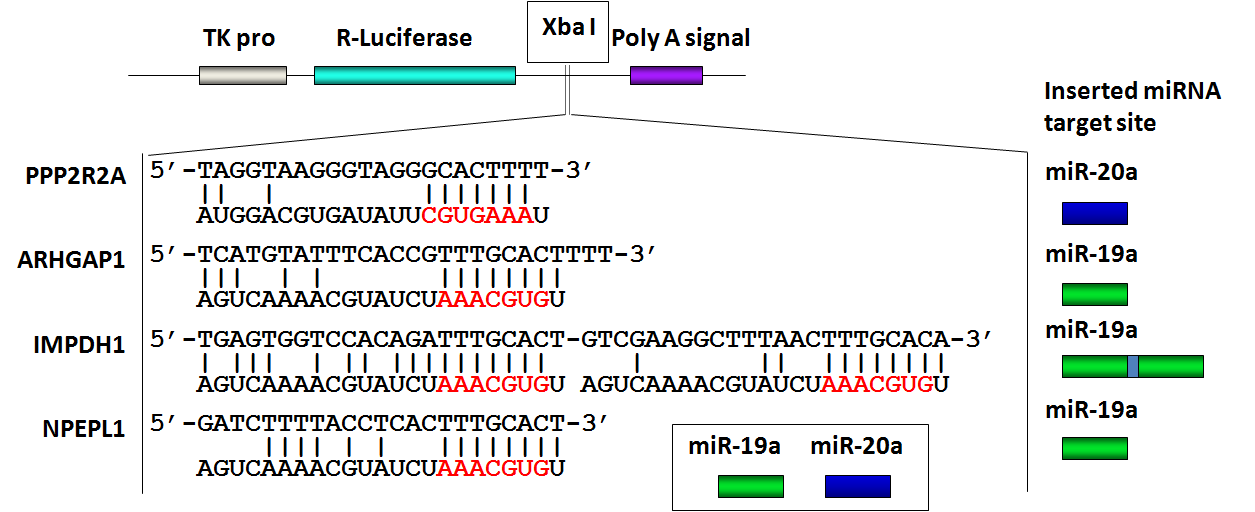

Supplement: Figure S4 — Construction of the luciferase vectors. The miR-17–92 target sites of the candidate genes were cloned downstream of the luciferase ORF at the XbaI restriction site of the pTK-hRG vector. The miRNA target sites are shown as nucleotide sequences (left) and as boxes (right). Sense (upper) and antisense (lower) strands of complementary sequences indicate the miRNA target site of mRNA 3′ UTR and the corresponding miRNA sequences, respectively. Seven nucleotides (red) on miRNAs show the seed sequences for binding with mRNA. (TIF) [file pone.0044095.s004.tif]
